# Supplementary material for: Sequence-specific targeting of GC-rich gene loci by parallel triplex-forming oligonucleotides containing a modified nucleobase
Source: Nucleic Acids Res. 2026 Oct 26;54(19):gkag998. doi: 10.1093/nar/gkag998 (PMC13696076; doi:10.1093/nar/gkag998)
Supplement: Supplementary file 1 [file gkag998_supplemental_file.docx]

**Supplementary Information**

**Sequence-Specific Targeting of GC-Rich Gene Loci by Parallel Triplex-Forming Oligonucleotides Containing a Modified Nucleobase**

Ruolin Ma,^1^ Michael Brazzill,^1^ Dylan Justice,^1^ Nicholas Buckham,^1^ Cen Chen,^2,3^ Shuichi Hoshika,^2,3*^ Steven A. Benner,^2,3^ and David A. Rusling^1*^

^1^School of Medicine, Pharmacy and Biomedical Science, University of Portsmouth, Portsmouth, PO1 2DT, UK

^2^Foundation for Applied Molecular Evolution, Alachua, FL 32615, USA

^3^Firebird Biomolecular Sciences LLC, Alachua, FL 32615, USA

*Corresponding authors: [david.rusling@port.ac.uk](mailto:david.rusling@port.ac.uk) and [shoshika@ffame.org](mailto:shoshika@ffame.org)

**Table S1** **| Oligonucleotide sequences used in this study.** The nucleobase 6-amino-5-nitropyridin-2-one (**Z**) under investigation is highlighted in bold. Underlined Ts in the P2 ZT-TFO-LNA oligonucleotide represent LNA-modified residues.

| **Target** | **Name** | **Sequence** |
| --- | --- | --- |
| P1 | Duplex R-strand | 5’AGGGTGGGGAGGGTGGGGAA |
| P1 | Duplex Y-strand | 5’TTCCCCACCCTCCCCACCCT |
| P1 | P1 GT-TFO | 5’TTGGGGTGGGTGGGGTGGGT |
| P1 | P1 GA-TFO | 5’AAGGGGTGGGAGGGGTGGGA |
| P1 | P1 GA-TFO-clamp | 5’AGGGTGGGGAGGGTGGGGAAttttAAGGGGTGGGAGGGGTGGGA |
| P1 | P1 CT-TFO | 5’TCCCGCCCCTCCCGCCCCTT |
| P1 | P1 ZT-TFO | 5’T**ZZZ**G**ZZZZ**T**ZZZ**G**ZZZZ**TT |
| P1 | P1 CT-TFO-clamp | 5’TCCCGCCCCTCCCGCCCCTTttttTTCCCCACCCTCCCCACCCT |
| P1 | P1 ZT-TFO-clamp | 5’T**ZZZ**G**ZZZZ**T**ZZZ**G**ZZZZ**TTttttTTCCCCACCCTCCCCACCCT |
| P1 | P1 sgRNA 1 template | 5’TTCTAATACGACTCACTATA**GAGGGTGGGGAGGGTGGGGA**GTTTTAGAGCTAGA |
| P1 | P1 sgRNA 2 template | 5’TTCTAATACGACTCACTATAG**CGCTTATGGGGAGGGTGGGG**GTTTTAGAGCTAGA |
| P1 | P1 sgRNA 3 template | 5’TTCTAATACGACTCACTATAG**AGGGGCGCTTATGGGGAGGG**GTTTTAGAGCTAGA |
| P1 | P1 sgRNA 4 template | 5’TTCTAATACGACTCACTATAG**TCCCCACCCTCCCCACCCTC**GTTTTAGAGCTAGA |
| P2 | Duplex R-strand | 5’GGAAAAAGAACGGAGGGA |
| P2 | Duplex Y-strand | 5’TCCCTCCGTTCTTTTTCC |
| P2 | P2 GT-TFO | 5’TGGGTGGTTTGTTTTTGG |
| P2 | P2 GA-TFO | 5’AGGGAGGTAAGAAAAAGG |
| P2 | P2-GA-TFO-clamp | 5’GGAAAAAGAACGGAGGGAttttAGGGAGGTAAGAAAAAGG |
| P2 | P2 CT-TFO | 5’CCTTTTTCTTTCCTCCCT |
| P2 | P2 ZT-TFO | 5’**ZZ**TTTTT**Z**TTT**ZZ**T**ZZZ**T |
| P2 | P2 ZT-TFO-LNA | 5’**ZZ**TTTTT**Z**TTT**ZZ**T**ZZZ**T |
| P1/P2 | ZT-TFO-scrambled | 5’T**ZZ**T**ZZ**T**ZZ**T**ZZ**T**ZZ** |
| P2 | P2 sgRNA template | 5’TTCTAATACGACTCACTATA**GCGGGAAAAAGAACGGAGGG**GTTTTAGAGCTAGA |
| qPCR primer | MYC  Forward | 5’CCTGGTGCTCCATGAGGAGAC |
| qPCR primer | MYC  Reverse | 5’CAGACTCTGACCTTTTGCCAGG |
| qPCR primer | GAPDH forward | 5’GTCTCCTCTGACTTCAACAGCG |
| qPCR primer | GAPDH reverse | 5’ACCACCCTGTTGCTGTAGCCAA |
| qPCR primer | β-Actin forward | 5’CACCATTGGCAATGAGCGGTTC |
| qPCR primer | β-Actin reverse | 5’AGGTCTTTGCGGATGTCCACGT |

**Figure S1** **| Possible W-C interactions of GT-, GA-, CT-, and ZT-containing TFOs with individual P1 duplex strands.** (a) Through G-C and mismatched base pairing, unmodified GT-, GA-, and CT-containing sequences could partner with either the pyrimidine (Y) strand or purine (R) strand of the P1 duplex, respectively. Mismatched base pairs are highlighted in bold. (b) By contrast, incompatibility of Z-G base pairing at neutral pH is unlikely to allow formation of the equivalent mismatched structures with the P1 pyrimidine (Y) strand. Mismatched base pairs are highlighted in bold.

**Figure S2** **| Targeting P1 and P2 with unmodified CT-containing TFOs requires low pH for triplex formation:** (a-b) EMSA analysis. Oligonucleotides were annealed in either pH 5.0 or pH 7.0 sodium cacodylate buffer containing 10 mM magnesium at final duplex concentration of 1 μM and either 10, 3, 1, 0.3, 0.1, or 0.03 μM TFO (lanes 2-7 of each gel, respectively). Complexes were then resolved on a 20% non-denaturing polyacrylamide gel in standard tris-acetate running buffer and visualised by post-staining with GelRed. Triple-stranded, double-stranded, and single-stranded species are indicated on the side of each gel by ts, ds, and ss abbreviations, respectively. Triplex formation was observed as a mobility shift relative to the duplex only control and was only observed at pH 5.0 for the unmodified CT-containing TFOs, consistent with the requirement for protonation of the C^+^:G-C triad. Interaction of the CT-containing TFO targeting P1 also resulted in additional higher order species, likely resulting from C^+^-C interactions between oligonucleotides.

**Figure S3** **| Targeting P1 and P2 sequences by TFOs is concentration dependant:** (a-b) EMSA analysis. Oligonucleotides were annealed in pH 7.0 sodium cacodylate buffer containing 10 mM magnesium at final duplex concentration of 1 μM and either 10, 3, 1, 0.3, 0.1, 0.03, or 0.001 μM TFO (lanes 2-8, respectively). Complexes were then resolved on a 20% non-denaturing polyacrylamide gel in tris-acetate running buffer and visualised by post-staining with GelRed. Triple-stranded, double-stranded, and single-stranded species are indicated on the side of each gel by ts, ds, and ss abbreviations, respectively. Triplex formation was observed as a mobility shift relative to the duplex only control and was concentration dependent for all TFOs. Interaction of the GA-containing TFO-clamp targeting P1 resulted in additional higher order species, most likely resulting from G-G or G-A interactions between oligonucleotides.

**Figure S4** **| Targeting P1 and P2 sequences by TFOs (side-by-side comparison):** (a-b) EMSA analysis. Oligonucleotides were annealed in pH 7.0 sodium cacodylate buffer containing 10 mM magnesium at final duplex concentration of 1 μM and 3 μM TFO (lanes 2-8, respectively). Complexes were then resolved on a 20% non-denaturing polyacrylamide gel in standard tris-acetate running buffer and visualised by post-staining with GelRed. Triple-stranded, double-stranded, single-stranded, and clamp species are indicated on the side of each gel by ts, ds, ss, and clamp abbreviations, respectively. Triplex formation was observed as a mobility shift relative to the duplex only control and each of the TFOs produced a triplex species that ran with a similar mobility, apart from the P1 CT-TFO clamp which exhibited a further reduction in mobility, likely reflecting partial binding of only the W-C part of the clamp at pH 7.0.

**Figure S5** **| sgRNA design and targeting of P1 and P2 sequences by Cas9 and SpRY Cas9. (a)** sgRNA targeting at P2. The sgRNA was designed to target the pyrimidine (Y) strand (nucleotides highlighted in yellow) of the TFO target sequence (underlined). **(b)** sgRNA targeting at P1. sgRNA were designed to target either the pyrimidine (Y) or purine (R) strand (coloured nucleotides) of the TFO target sequences (underlined). Note: P1 sgRNA 4 lacks a compatible PAM sequence but was expected to work efficiently with PAM-independent SpRY Cas9. **(c)** Cas9 and SpRY Cas 9 protection assay at P1. 30 nM pMYC plasmid was prepared in NEBuffer 3.1 buffer. The Cas9/sgRNA complex was then incubated with samples at 37 °C for 1 hour. Following inactivation, cleavage products were resolved on a 0.8% agarose gel and visualized by GelRed staining. Cas9-mediated cleavage was revealed by conversion of the supercoiled plasmid to its linear form and greater efficiency was observed using PAM-relaxed SpRY Cas9. Of note, neither sgRNA 2 nor sgRNA 3 that target sequences adjacent to the quadruplex-forming P1 sequence could direct cleavage to the underlying DNA.

**Figure S6** **| dCas9 control experiments.** 100 ng plasmid DNA was pre-incubated with 67 nM of either dCas9 alone, sgRNA alone, or the full dCas9/sgRNA complexes in DMEM buffer prior to co-transfection using Lipofectamine 3000^TM^. After 24 hours, luciferase activities were quantified using a SpectraMax i3x Microplate Reader. Activities were normalized to a vehicle treated dH_2_O control. Data represent at least three independent biological replicates. Statistical significance was assessed by one-way ANOVA with Tukey’s multiple-comparisons test. Error bars indicate mean ± s.d. Only the dCas9/P2 sgRNA complex resulted in a reduction in luciferase activity relative to control sample.

**Figure S7** **| HEK293 viability in presence of P1 and P2 oligonucleotides.** 100 ng plasmid DNA was pre-incubated with 67 nM TFO or Cas9/sgRNA in DMEM buffer prior to co-transfection using Lipofectamine 3000^TM^. After 24 hours post-transfection, 8 μl PrestoBlue™ reagent was added directly to each well and incubated for 30 min at 37°C. Fluorescence was then measured using a SpectraMax i3x microplate reader (Molecular Devices) with excitation at 560 nm and emission at 590 nm. Experiments were performed with a minimum of three biological replicates and viability values were normalised to the vehicle control (dH₂O). Statistical significance was assessed by one-way ANOVA with Tukey’s multiple-comparisons test. Error bars indicate mean ± s.d. None of the oligonucleotides, nor dCas9, resulted in a statically significant decrease in cell viability at the timepoint and oligonucleotide concentrations tested.

**Figure S8** **| Relative *MYC* mRNA levels in various cell lines.** *MYC* RNA levels were quantified 24 h after transfection and quantified by qPCR. Total RNA was extracted, reverse transcribed, and analyzed by SYBR Green qPCR with primers specific for MYC, GAPDH and *β-actin*. Relative *MYC* RNA levels were normalised to *GAPDH* and *β-actin* then to the dH_2_O vehicle control. Of the cell lines tested, HCT116 cells exhibited the highest level of *MYC* transcription.

**Figure S9** **| Site-specific repression at P1 and P2 in the endogenous MYC promoter in HCT116 cells (western blot analysis). (a-b)** Oligonucleotides were first diluted in Opti-MEM buffer to final concentrations of 2500, 250 and 25 nM (indicated by triangle), complexed with Oligofectamine^TM^ reagent in a total volume of 200 μl, and added to HCT116 cells. Endogenous MYC protein levels were quantified 48 h after transfection by western blotting. Cells were lysed in RIPA buffer supplemented with protease and phosphatase inhibitors, and total protein concentration was determined by BCA assay. Equal amounts of protein were resolved by SDS-PAGE, transferred to nitrocellulose membranes, and probed with antibodies against MYC and GAPDH. Signals were detected using a LI-COR Odyssey CLx imager and quantified in ImageJ. Relative MYC protein levels were normalised to GAPDH and then to the dH_2_O vehicle control. Data represent a minimum of two biological replicates; statistical significance was determined by one-way ANOVA with Tukey’s post hoc test. Triplex formation is indicated by a reduction in MYC protein levels relative to control sample.

**Figure S10** **| HCT116 viability in presence of P1 and P2 oligonucleotides.** 2,500 nM of oligonucleotides were incubated with cells in Opti-MEM buffer prior to transfection using Oligofectamine^TM^ reagent. After 24 hours post-transfection, 8 μl PrestoBlue™ reagent was added directly to each well and incubated for 30 min at 37°C. Fluorescence was then measured using a SpectraMax i3x microplate reader (Molecular Devices) with excitation at 560 nm and emission at 590 nm. Experiments were performed with a minimum of three biological replicates and viability values normalised to the vehicle control (dH₂O). Statistical significance was assessed by one-way ANOVA with Tukey’s multiple-comparisons test. Error bars indicate mean ± s.d. None of the oligonucleotides resulted in a statically significant decrease in cell viability at the timepoint and oligonucleotide concentrations tested.
